# Designing a Smart Garment for Dynamic Sitting Reminders

**DOI:** 10.3390/s25113359

**Published:** 2025-05-27

**Authors:** Yujie Hou, Zhaohui Wang, Huanhuan Liu, Mengxuan Xia, Xinyi Fan, Qinwen Ye

**Affiliations:** 1 College of Fashion and Design, Donghua University, Shanghai 200051, China; 2221733@mail.dhu.edu.cn (Y.H.); hhliu@mail.dhu.edu.cn (H.L.); 19979905331@163.com (M.X.); 18268007210@163.com (X.F.); yeqinwen_20121220@163.com (Q.Y.); 2 Key Laboratory of Clothing Design and Technology, Donghua University, Ministry of Education, Shanghai 200051, China

**Keywords:** textile sensors, smart garment, dynamic sitting, machine learning, feedback mode

## Abstract

**Highlights:**

**What are the main findings?**

- We designed a textile sensor-based smart garment that enables sitting posture monitoring, recognition, and feedback while balancing functionality, comfort, and aesthetics.
- Since dynamic sitting is healthier than maintaining a rigid upright posture, we have developed a “Dynamic Sitting” feedback system for office workers.

**What is the implication of the main finding?**

- The design of this smart clothing can increase the user’s willingness to buy and use the product.
- The “Dynamic Sitting” reminder system can effectively modify users’ sitting habits without work disruption, thus reducing the risk of sedentary-related diseases.

**Abstract:**

Currently, the sedentary nature of office work has led to a steady increase in the prevalence of spinal disorders, including lower back pain, back pain, and neck pain. Medical research has shown that monitoring and improving sitting posture is an important measure to prevent spinal discomfort. The emergence and development of wearable technology have enabled more people to effectively monitor their health. In this study, we propose and design a textile sensor-based sitting posture correction smart garment to realize dynamic sitting reminders aimed at meeting the needs of sedentary office workers. The garment achieves real-time sitting posture recognition through integrated machine learning algorithms, with a recognition accuracy exceeding 95% using a random forest classifier. Additionally, we developed haptic vibration feedback and visual GUI feedback modes to provide sitting posture intervention and dynamic sitting reminders. To evaluate the system’s effectiveness and usability, we conducted comparative experiments analyzing sitting posture behavior before and after wearing the smart garment, along with a user satisfaction survey. The results demonstrate that the smart garment effectively helps office workers adjust their sitting posture and reduces the risk of spinal discomfort associated with prolonged sedentary work.

## 1. Introduction

The high-quality development of mobile Internet of Things (IoT) technology has expanded the scale of network user groups, making network-based office work the mainstream mode in the current era. However, while enjoying the convenience brought by mobile office solutions, the sedentary work style has increasingly posed health risks to a growing number of office workers. Sedentary work, as a low energy-consuming activity, has been proven to cause a series of health problems, including obesity, diabetes, cardiovascular diseases, and musculoskeletal disorders (MSDs) [1]. Among these, musculoskeletal disorders are the most common health issues affecting the large occupational group of sedentary office workers [2,3]. Data show that 25–51% of sedentary office workers experience symptoms of low back pain and neck or shoulder pain, with low back pain being the most common [4,5]. In addition to sedentary time, different postures impose different compressive loads on the lumbar spine. Maintaining a particularly poor sitting posture for a long period of time can also increase the risk of spinal disorders due to an imbalance of muscular forces [6,7,8]. Therefore, early monitoring and intervention are particularly important. Distinguished from the traditional passive adjustment of orthopedic belts, if the product can guide people to self-correction from subjective awareness through monitoring recognition and feedback, it will open up new ways and possibilities for the prevention of spinal health disorders.

Currently, researchers have proposed various technological approaches to the problem of sitting posture monitoring and recognition, which are mainly categorized into three groups: machine vision techniques, pressure sensors, and wearable sensors [9,10]. Machine vision techniques rely on cameras and are affected by ambient lighting conditions, while pressure sensors depend on seats, both of which are constrained by usage scenarios and lack convenience [11,12]. With the development of flexible and intelligent wearable devices, wearable sensing technology has been gaining more and more attention and has shown significant value in areas such as medical rehabilitation and human–computer interaction [13]. More and more studies have integrated wearable sensing technologies into garments for real-time monitoring and classification of sitting postures, and these garments have demonstrated high accuracy, reliability, and convenience. This is attributed to the inherent unity of movement between garments and the human body, as well as the garment system’s ability to intuitively capture changes in the user’s spinal column and back muscles during different sitting postures [14]. Garment systems designed for sitting posture health monitoring must address the technical challenges of integrating electronic components with fabrics, particularly concerning flexibility, comfort, and aesthetics. Furthermore, the effectiveness of feedback mechanisms for posture correction, including their applicability to the current context and their ability to help users improve sitting behavior, remains a crucial issue that requires further resolution.

The aim of this study is to develop a sitting posture correction smart garment designed for sedentary office workers, helping users achieve dynamic sitting posture reminders. This paper makes the following contributions. (1) In garment design, we integrate textile sensors into the garment for sitting posture monitoring, ensuring flexibility and comfort. At the same time, we optimize the design of the garment structure to achieve effective integration of sensors and enhance the aesthetics of the garment. (2) In terms of system functionality, we focus on real-life scenarios of sedentary office users. By incorporating the concept of “Dynamic Sitting”, we propose a vibration feedback mode to help users realize dynamic sitting reminders so as to adjust their sitting behavior. Different from the traditional feedback mode that emphasizes maintaining an upright sitting posture, the feedback mode proposed in this paper is more in line with the natural postural changes of the human body, which is more reasonable. In addition, the GUI interface displays the sitting status in real time, which enhances the interactivity between the user and the garment system. The combination of tactile and visual multi-sensory interaction can better meet the application requirements of this type of smart garment. We expect that our study will provide valuable insights and references for the development of sitting health monitoring products for sedentary office workers.

## 2. Related Work

In the current research on clothing systems for sitting posture monitoring, the most commonly used wearable sensors are still inertial sensors [15]. Bootsman [16] developed a posture-monitoring smart garment for nurses in the workplace called Backup, which embeds two inertial measurement units (IMUs) to be placed at lumbar spine L1 and L5 to monitor lumbar flexion changes to recognize sitting posture. However, inertial measurement units have a certain degree of rigidity and stiffness, which can affect the comfort of the wearer when embedded in garments [17]. Moreover, in terms of posture recognition, the inertial sensor only monitors the curvature of the human spine. However, when the posture changes, the stretching of the back muscles can also affect the accuracy of the measurement. For instance, when the user adopts a sitting posture with crossed legs, the spine angle may not change significantly. Relying solely on the inertial sensor makes it difficult to correctly identify the sitting posture category. In contrast, textile sensors can cover other muscle-stretching areas of the back, which can effectively improve the accuracy and comprehensiveness of posture recognition. At the same time, textile sensors are becoming a hotspot in smart wearable technology due to their flexible nature, allowing them to fit more closely to the human body and provide a comfortable wearing experience [18]. Currently, only a few scholars have utilized textile sensors to develop sitting posture monitoring garment systems for posture recognition and classification. Drashti Sikligar et al. [19] designed and fabricated textile capacitive and resistive sensors, which were sewn into garments to monitor daily changes in the lumbar and thoracic vertebrae during poor sitting postures. This system achieved sitting posture classification and provided valuable data for users’ sitting health research. Jiang et al. [20] proposed a self-powered sitting posture monitoring T-shirt based on triboelectric nanogenerators (TENGs). The sensors were integrated into the garment through sewing and positioned in multiple areas, including the cervical, thoracic, scapular, and lumbar regions, for posture data collection. Although both garments attempted to apply textile sensors, their aesthetic appeal remains lacking, and the structure of the sensors embedded in the garments still needs to be optimized and improved. At present, research on the application of textile sensors in garments for sitting posture monitoring remains relatively limited [14]. Flexibility is undoubtedly the primary condition for enhancing comfort in future smart garments for sitting posture correction, while at the same time, the integration relationship between textile sensors and garments needs to be considered comprehensively to improve the aesthetics of the garments.

In addition, in the current existing smart garment system for sitting posture correction in the feedback mode, there are still some problems, especially in the sedentary office scenarios where applicability is poor. For example, Li [21] designed a set of smart posture correction garment systems, which reminds the user to maintain an upright sitting posture through vibration and an embedded buzzer when there is a bad sitting posture. However, the buzzer sound prompt is not suitable for use in public places, which limits the practical application of the feedback mode. Ferdews Tlili [22] designed a real-time monitoring orthopedic belt system for bad sitting posture, along with a supporting cell phone app. When the user logs into the system and the system detects bad sitting posture, the cell phone will receive a screen notification and sound alerts. This type of visual and sound reminder is not applicable in office scenarios, as users cannot always pay attention to cell phone messages while working. Therefore, the feedback mode needs to be designed with due consideration to the characteristics of public office scenarios so that it can effectively remind the user without interfering with the work of people around them [23]. Another critical point is that previous feedback mechanisms have primarily aimed to encourage users to maintain an upright sitting posture [12]. However, research has shown that this approach is not entirely reasonable. Any prolonged static sitting posture can lead to fatigue, discomfort, and pain, which means that maintaining an upright sitting posture for an extended period without interruption is still harmful [24,25,26,27]. Academics have introduced the concept of “dynamic sitting”, which involves frequent changes in body position while seated [28,29], as an effective strategy to mitigate the health risks associated with prolonged static sitting. Therefore, it is necessary to incorporate the concept of “dynamic sitting” into the optimal design of the feedback mode.

Although relevant studies have demonstrated the accuracy of textile sensors in sitting posture monitoring and successfully realized feedback alerts for poor sitting posture, the aesthetics and practicality of the garment have not been fully considered, and little attention has been paid to the advantages of “dynamic sitting”. In this paper, we design and develop a smart garment system for sitting posture correction to address these needs. We will introduce the design of the smart garment system and describe how it enables posture monitoring, recognition, and dynamic sitting reminders. Finally, we evaluate the overall effectiveness of the system.

## 3. Design of the Garment System

The aim of this section is to build a complete smart garment system by analyzing the design requirements for subsequent sitting posture acquisition, recognition, and feedback.

### 3.1. Design Requirements

The design requirements for the smart garment system for sitting posture correction include wearing comfort, monitoring accuracy, and timely feedback. The details are described as follows. First, the garment system should be comfortable for people to wear and should be easy to put on and take off without restricting body movement. Second, the garment system needs to be able to correctly recognize and classify different sitting postures. To achieve this, it needs to make sure that the sensors are in the proper position on the garment and that the garment fits closely to the body to avoid slippage of electronic components, such as sensors. Finally, in terms of feedback mode, the garment system needs to be fully integrated with the concept of “dynamic sitting”, and a feedback mode suitable for sedentary office workers needs to be set up. In other words, when the user maintains a certain sitting posture for more than a predetermined period of time, the garment system will provide feedback to the user to inform the user to adjust the sitting posture. Furthermore, Yang et al. [30] demonstrated that users experience significantly increased discomfort after 45 min of sitting, necessitating feedback mechanisms to prompt movement at this interval.

It should be noted here that the feedback mode needs to be easy to understand and should not interfere with the user or those around them in the workplace. Obviously, sound-based reminders are clearly inappropriate in such scenarios. In contrast, tactile vibration feedback targets the user’s sense of touch, with adjustable vibration intensity and frequency, and will not disturb others in public spaces. Additionally, visual interface feedback allows users to intuitively understand their sitting posture and provides a convenient way to review data records of their sitting habits. Therefore, a combination of haptic and visual feedback is chosen for its effectiveness and suitability.

### 3.2. System Overview

**Figure 1:**
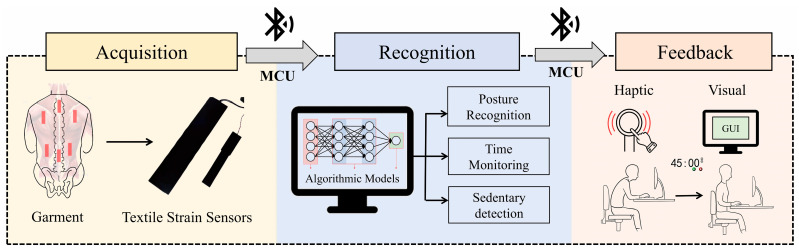
Architecture diagram of the garment system [12].

The overall architecture of the sitting posture correction smart garment system consists of three main layers: the acquisition layer, the recognition layer, and the feedback layer (as shown in Figure 1 [12]. The acquisition layer is used to process the data collected by the textile sensors by the microcontroller and transmit the data to the host computer program using Bluetooth communication devices. The recognition layer is where the collected data are analyzed by the machine learning model within the host computer program to generate the posture recognition results. In the feedback layer, the recognition results are directly displayed in real time through the GUI interface of the host computer program. Simultaneously, the results are transmitted via Bluetooth to the controller, which activates the vibration modules for haptic feedback.

### 3.3. Smart Garment Design

Smart garments for sitting posture correction mainly include the design of electronic modules, as well as the design and production of the garment, while ensuring the effective integration of the electronic system with the garment.

#### 3.3.1. Sensor Locations

In this smart garment system, sensors play a crucial role in real-time monitoring of the wearer’s posture. The accuracy of posture monitoring depends significantly on the placement of the sensors throughout the garment system. Considering that the principle of textile strain sensors is to generate electrical signals through tensile deformation, they need to be placed in areas where the human skin undergoes the most significant changes during different sitting postures. For this reason, we explored and determined the optimal placement of the sensor through a combination of theoretical analysis and experimental testing.

First, by analyzing the anatomy of the human back, we found that different sitting postures have different effects on various parts of the body, and changes in sitting posture cause changes in spinal curvature and muscle stretching and contraction. Muscle changes are mainly concentrated in the mid-thoracic back region and the back region of the transition between the thoracic and lumbar regions. Specifically, the back muscles with greater deformation are the middle trapezius muscle, lower trapezius muscle, rhomboid, and erector spinae muscles.

Further, we conducted experimental tests on the deformation rate of human skin in different sitting postures. Several typical sitting postures were selected, including sitting upright, sitting with one’s back bent, leg sitting (both left and right), sitting forward, and backward-leaning sitting. A grid was drawn on the back of the human body based on the dimensions of key body parts. The body surface was mapped according to this grid, and 10 users were selected for 3D body scanning in different sitting postures. After exporting the data, the deformation rate of human skin was calculated.

**Figure 2:**
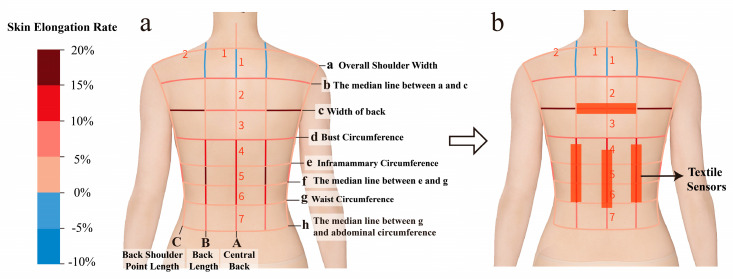
Determination of sensor placement position: (**a**) skin deformation grid lines in different sitting postures; (**b**) sensor location.

To intuitively reflect the change in local skin size, we used the human skin change grid to display the data. Using a grading method, we assigned values to the intervals. Red represents the stretch rate and blue represents the contraction rate, and the darker the color, the higher the stretch or contraction rate. The results are shown in Figure 2a, which shows that in the transverse dimension, the region at the dorsal breadth line (i.e., the real length of the body surface from the right posterior axillary point to the left posterior axillary point) has the greatest change, which roughly corresponds to the level of thoracic vertebrae T5–T7 and involves the erector spinae muscle and the lower trapezius muscle mainly. The contraction and stretching of the erector spinae muscle would lead to significant deformation of the skin surface during changes in sitting posture; meanwhile, the elongation or contraction of the lower trapezius muscle, an important muscle connecting the scapula to the spine, also causes changes in the rate of skin deformation. This experimental conclusion aligns with the theoretical analysis. In the longitudinal dimension, the dorsal region at the transition between the thoracic and lumbar regions experienced the most change. This region roughly corresponds to the levels of thoracic vertebrae T11–T12 and lumbar vertebrae L1–L3, which is the key area where the spine transitions from thoracic kyphosis to lumbar lordosis. It is also where the physiological curvature of the spine changes significantly. The contraction and stretching of core muscles, such as the erector spinae and quadratus lumborum, during changes in sitting posture, lead to a significant increase in the rate of skin deformation, further supporting the validity of the experimental findings. Therefore, the four sensors were placed in the region with the largest skin deformation, as shown in the red rectangular region in Figure 2b. Compared to existing sitting posture monitoring garments, this system benefits from more objective and data-driven evidence for sensor placement, greatly enhancing its reliability.

#### 3.3.2. Electronic Modules

**Figure 3:**
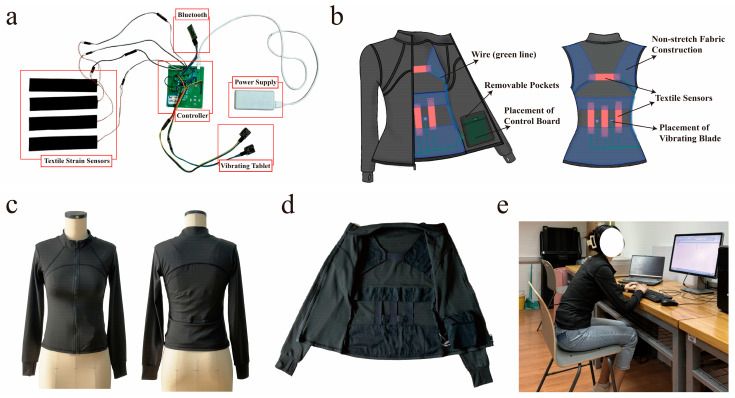
Design and realization of the smart garment: (**a**) diagram of electronic module connections; (**b**) garment internal structure and detail design; (**c**) presentation of ready-to-wear garments; (**d**) demonstration of the internal structure of the garment; (**e**) figure for data collection wearing the garment.

The sitting posture correction smart garment system consists of an electronic system module and a clothing part. The electronic module is composed of the following components: (a) four textile strain sensors, a size of 150 mm × 30 mm, a strain resolution of 0.05%, and a linearity of 99.9%. The sensor is produced by the Elastech company (Ningbo, China) and can measure the strain deformation of the human body at specific locations and output capacitance values [31]. (b) An STM32 (model Geehy series E103VET6) microcontroller (developed by Positive Point Atomics, Guangzhou, China) serves as the core of the system. Since the STM32 can only recognize resistive signals, it is paired with the PCAP01 Capacitance-to-Digital Converter SoC (developed by MincoCore Electronics, Shenzhen, China) to convert capacitance data monitored by the sensor. (c) An HC-05 Bluetooth module (developed by Telesky, Shenzhen, China) facilitates communication between the system and the host computer. (d) Two vibration patch motors (developed by Telesky, Shenzhen, China) measuring 21 mm × 23 mm are used to provide vibration feedback. (e) A battery (developed by Zave, Jinhua, China) supplies power to the system. The overall connection of the electronic module is illustrated in Figure 3a.

#### 3.3.3. Garment Structure Design

The garment design in this study is primarily targeted at women and takes into account both aesthetic and functional requirements, as follows.

In terms of appearance, the garment should have a simple, everyday design that is suitable for multiple scenarios, such as daily commuting and sports activities. Additionally, to ensure the stability of data collection, the sensors need to maintain close contact with the skin. Therefore, the garment is designed as tight-fitting elastic wear. The specific appearance of the clothing design is shown in Figure 3b.

In terms of functionality, the first consideration is the sensing function. To ensure that the sensor can stretch and deform at the pre-set position, a non-elastic inner structure is added inside the garment to counteract the elasticity of the tight-fitting jacket, which might otherwise affect the deformation of the textile sensor. As shown in the blue areas in Figure 3b, this inner structure is connected to both sides of the sensor, facilitating the sensor’s stretching. The second consideration is the vibration feedback function. The placement of the vibration components is crucial—they should be located on muscles and positioned where they can effectively prompt the user to adjust their posture. Consequently, the vibration components are placed on the erector spinae muscles on both sides of the spine, just above the waistline, as indicated by the yellow areas in Figure 3b.

#### 3.3.4. Functional Integration

The electronic module circuits shown in Figure 3a are integrated into the garment, as illustrated in Figure 3b. The sensors are sewn into the garment’s internal non-elastic structure, the circuitry is concealed within the inner and outer layers of the fabric, and removable pockets are incorporated on the inside specifically for components, such as controller panels, Bluetooth, and batteries, to try to balance the garment’s aesthetics and comfort. The fully integrated garment is shown in Figure 3c, with its internal structure displayed in Figure 3d. The overall circuits are detachable so that when the user needs to exercise, the electronic components can be removed to meet the user’s needs for multi-scenario wear.

## 4. Sitting Posture Recognition and Feedback

To implement the dynamic sitting reminder function, this section will use the designed smart garment to collect sitting signals and pre-process the data, compare multiple machine learning algorithms, and filter out the optimal sitting classification recognition model. At the same time, combined with the concept of “dynamic sitting”, tactile and visual sensory feedback modes will be designed to enable real-time monitoring and dynamic reminders of sitting posture, helping sedentary office workers improve their sitting behavior.

### 4.1. Experiments on Sitting Signal Acquisition

The purpose of this experiment is to obtain signal data from users with different sitting postures in order to provide a training dataset for the subsequent construction of a sitting recognition classification model. Notably, our experiment received ethical approval from the Human Research Ethics Committee of Donghua University. Detailed information about the experimental procedures and the purpose of this study was provided to each participant, who voluntarily signed a research consent form.

#### 4.1.1. Participants

Twenty female participants were recruited for this experiment, with the following inclusion criteria: (a) a height of 160 ± 5 cm, a weight of 48 ± 3 kg, and the ability to wear the experimental garment; (b) regular long-term use of a computer for office work; and (c) good overall health, with no musculoskeletal disorders. Moreover, during the experiment, the participants were required to wear a close-fitting undershirt on their upper body beforehand to prevent any interference from their garment with experimental clothing.

#### 4.1.2. Process

In this paper, the data from four sensors are used to recognize sitting posture. Since the muscle stretching changes differ in each posture, the threshold ranges monitored by the corresponding four sensors also vary for each posture.

**Figure 4:**
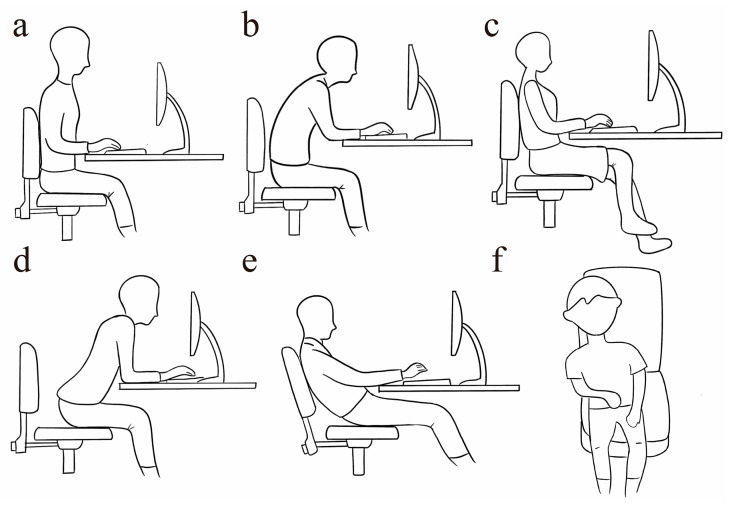
Selection of target sitting position: (**a**) sitting upright; (**b**) sitting with one’s back bent; (**c**) leg crossed; (**d**) sitting forward; (**e**) leaning backward; (**f**) other sitting postures.

Before the experiment began, first, the types of postures to be captured were determined, including upright sitting, sitting with one’s back bent, leg crossed (left and right leg crossed), sitting forward, leaning backward, and other sitting postures (e.g., tilting), as illustrated in Figure 4. In addition, since the system is designed to provide sedentary reminders, it is necessary to capture signals from standing postures to differentiate between sitting and standing states in subsequent analyses.

The participants were then allowed to wear the experimental garment and complete the acquisition of the target sitting postures, as shown in Figure 3e. Each sitting posture was maintained for 30 s, and the participants were required to perform the tasks of typing and editing documents during the acquisition process. The controller’s acquisition frequency was set to capture data twice per second. As a result, each participant could collect approximately 60 groups of data samples for each posture, totaling around 480 groups of data samples per participant. In total, about 9600 data samples were obtained.

#### 4.1.3. Data Analysis

**Figure 5:**
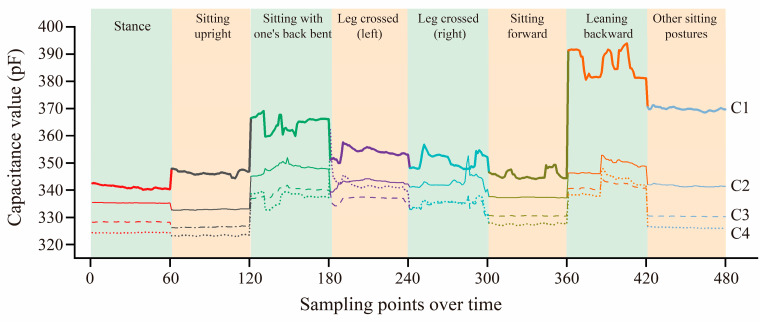
Comparison graph of acquired signal data for the different poses.

**Figure 6:**
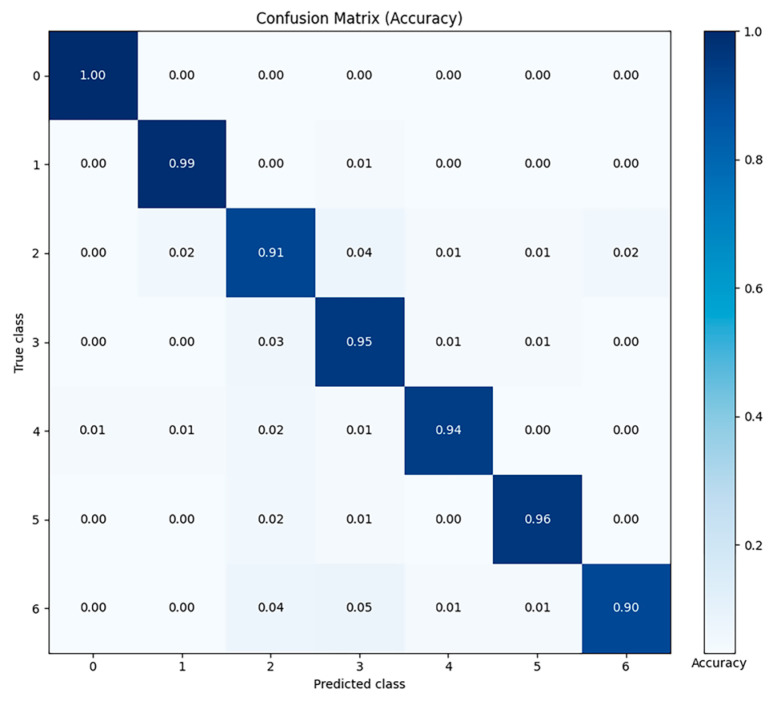
Data validation results for the random forest model (0: stance, 1: sitting upright, 2: sitting with one’s back bent, 3: leg crossed, 4: sitting forward, 5: leaning backward, 6: other sitting postures).

In order to further verify that the signals collected by our designed garment system are valid for sitting posture classification and can reflect the trend of changes in each sensor across different sitting postures, we randomly selected data from one user in various sitting postures for comparative analysis, as shown in Figure 5. Among these, by comparing the signal characteristics under different sitting postures, it is clearly observed that there is significant variability in the signal thresholds of different sensors across postures, and the capacitance signal value of sensor C1 in the center region at the dorsal width line changes most significantly (the thick solid line labeled C1 in Figure 6). However, it is worth noting that the difference in signal characteristics between the two sitting postures, left leg crossed and right leg crossed, is relatively small and not easy to differentiate using data from these four sensors alone. Therefore, the left leg crossed and right leg crossed sitting postures were combined into the leg crossed sitting posture in the subsequent classification problem.

#### 4.1.4. Data Pre-Processing

The collected samples undergo data pre-processing. First, the sample data are checked for validity, and any invalid data samples are eliminated. This step helps reduce the bias introduced by anomalous data during subsequent model training, ensures the quality of the dataset, and improves the accuracy and reliability of the classification model. Next, the dataset is constructed. Since the system is designed to classify and recognize seven selected types of postures, the system needs to use a supervised machine learning algorithm to categorize the sitting poses and needs to manually encode a numerical label for each pose. As described in Section 4.1.2, the selected postures in this study include upright sitting, sitting with one’s back bent, leg crossed (left and right leg crossed), sitting forward, leaning backward, and other sitting postures, so the above postures are encoded as 0, 1, 2, 3, 4, 5, and 6, respectively. Considering that differences in users’ height and weight during data collection can result in variations in the signal amplitudes measured for the same sitting posture, the collected samples are normalized using MinMaxScaler. This normalization process helps eliminate classification errors caused by individual differences and improves the accuracy of the classification model.

### 4.2. Sitting Position Recognition

#### 4.2.1. Recognition Methods

In order to effectively recognize and classify different types of sitting postures, it is necessary to establish a correlation model between human sitting postures and electrical signals. And, machine learning can build accurate mathematical mapping functions based on iterative learning and training of large samples so that sitting postures can be effectively recognized and predicted by well-trained models [20]. This study focuses on classifying sitting postures, which is essentially a multi-classification problem. The model needs to predict the corresponding sitting category based on the electrical signal data input from the sensors, so selecting an appropriate machine learning algorithm is crucial. In this study, four commonly used classification algorithms were selected for comparison and evaluation: K-nearest neighbor (KNN), support vector machine (SVM), decision tree, and random forest. These algorithms are extensively utilized in machine learning, each possessing distinct advantages for specific applications [32].

#### 4.2.2. Recognition Results

In this study, 80% of the dataset was used for model training, while the remaining 20% was reserved for model testing and performance evaluation. And, in order to find the most optimized model parameter configuration in the selection of each model parameter, this study adopts the Grid Search (GSE) algorithm to systematically search and evaluate the effect of the combination of different parameters. This system has trained and compared four models, K-nearest neighbor, support vector machine (SVM), decision tree, and random forest, obtained the optimal parameters of each model, and counted the accuracy statistics of each model, as shown in Table 1. From the comparison of the data in Table 1, it can be found that, except for the decision tree model, which has a slightly lower accuracy rate and fails to reach 90%, all the other models have an accuracy rate higher than 90%, showing good classification performance. It is especially noted that the random forest training model obtained the highest prediction accuracy of more than 95% in this experiment.

In order to further evaluate the optimal algorithm, the system was re-analyzed using the ten-fold cross-validation method, and the results are shown in Table 2. From the results, it can be seen that the K-nearest neighbor algorithm also shows good performance and strong stability, while the support vector machine also achieves fairly high accuracy but with a relatively high standard deviation, indicating poor stability. Additionally, during the model training and validation process, the training time for the support vector machine is much longer than that of other models. In the comprehensive analysis, the random forest model has the highest accuracy and the strongest stability, so this system ultimately selects the random forest as the classification model for sitting posture recognition.

In order to demonstrate more intuitively the classification performance of the random forest model in the sitting recognition task, we used the Confusion Matrix to visualize the relationship between the predicted results of the model and the actual labels. The results of the predicted and true values for each sitting posture are shown in Figure 6.

### 4.3. Design of Feedback Mode

The aim of this study is to develop a system that helps sedentary users achieve “dynamic sitting posture” reminders. Therefore, after recognizing the sitting posture, it is necessary to design an effective feedback mode to help users adjust their sitting posture and enable active intervention. Considering the work environment and behavioral characteristics of office workers, the feedback mode of this system is designed with tactile feedback as the primary method and visual feedback as a supplementary option.

#### 4.3.1. Haptic

Haptic feedback is a way of direct contact with the human body and also serves as a medium for emotional expression. In the sitting posture correction system, the most commonly used haptic reminder is vibration feedback. The vibration and its intensity are directly transmitted to the skin’s sensory layer so that it can effectively play a reminder of the role.

Combined with the concept of dynamic sitting, what we aim to achieve is to remind the user to change their sitting posture after maintaining the same posture for a certain period of time. However, the degree of spinal curvature and muscle deformation caused by different sitting postures are not the same, and thus the degree of harm to health is not the same. According to physiological studies of sitting posture [33], leg crossed is the most harmful, followed by sitting with one’s back bent, leaning backward, and sitting forward, with sitting upright being the least harmful. Furthermore, based on recommendations from musculoskeletal experts, office workers should change their sitting posture every 15–20 min, with leg crossed requiring a change every 5−10 min [34,35]. Therefore, when setting the vibration mode, the system divides the sitting postures into three levels according to their degree of harm, and each level has different time intervals for reminding the change in sitting postures. The specific vibration reminder modes for “Dynamic Sitting” are shown in Table 3. In the actual vibration motor reminder program setting, the logic of the program uses the counting method. Every second, it records the current sitting posture category. As time increases, when the time reaches 5 min, the system traverses all recorded data. Every 5, 10, and 15 min, the number of each posture is counted. Considering the accuracy of machine learning and the small movements during sitting that may lead to error results, a threshold of 70% accuracy is set. Specifically, for the 5 min statistics, if more than 70% of the recorded postures are the leg crossed sitting posture, it is recognized that the user has maintained the leg crossed sitting posture for 5 min, triggering the vibration reminder.

To provide users with a clearer and more comfortable vibration perception experience, this study conducted a user preference experiment to explore optimal vibration frequency and time. Twelve office women were selected to wear the experimental garment with vibration patches, simulating real work scenarios. They felt the vibrations of the patches at different frequencies, as well as the vibration of the same frequency at different times. At the end of the experiment, the participants rated the comfort of the vibration frequency and time. The subjective ratings were based on a five-point Likert scale, with scores ranging from 1 to 5.

**Figure 7:**
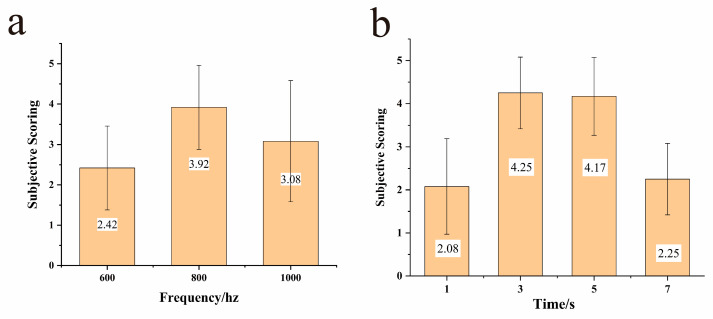
Subjective comfort score: (**a**) vibration frequency; (**b**) vibration duration.

The experimental results are shown in Figure 7, where it can be observed that the frequency of 800 Hz and the time of 3 s has the highest score. Therefore, the system ultimately selects the vibration mode with a frequency of 800 Hz and a vibration time of 3 s to help users change their sitting posture. To distinguish this from the sedentary reminder at 45 min, and to remind obviously the user to stand up and move, in particular, the vibration time for the sedentary reminder is set to 7 s.

#### 4.3.2. Visual

**Figure 8:**
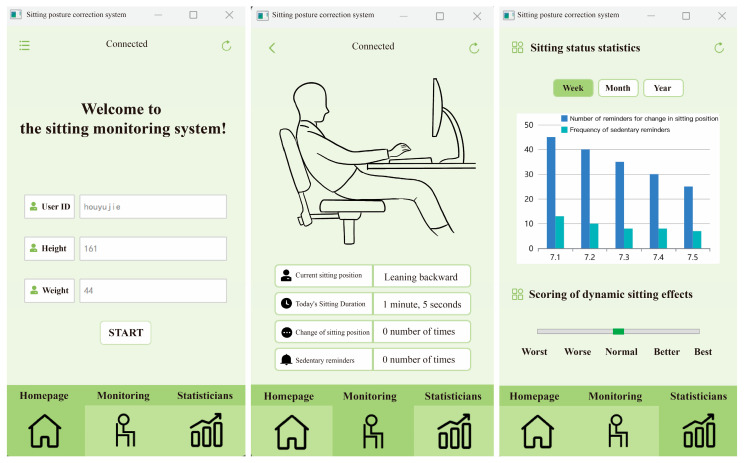
GUI interface demonstration.

Visual feedback is one of the most direct ways to obtain information, which can help users perceive more detailed features. To enable users to understand their current sitting posture more intuitively and clearly, we designed a Graphical User Interface (GUI). When the user starts the host computer program to run the sitting posture recognition code, the GUI automatically opens and displays the user’s sitting status in real time. It is important to note that sedentary office workers may not always be able to monitor the changes on the interface due to their busy schedules. Therefore, the interface also includes a series of data logging functions related to the user’s sitting posture, including the total sitting duration for the day, the number of times the user changed their sitting posture, and the number of sedentary reminders received. Moreover, to help users better track their sitting behavior changes, we also designed a history module in the GUI interface. This allows previous data to be stored as historical records, making it easier for users to compare sitting behavior before and after wearing the garment, which provides valuable reference data for future sitting management. The specific GUI interface design is shown in Figure 8.

## 5. Garment Evaluation Tests

Through behavioral intervention effectiveness experiments and user satisfaction surveys, combined with objective data and subjective ratings, this paper provides a comprehensive assessment of the system’s utility. Ethical approval for this study was obtained from the Ethics Committee of Donghua University (SRSY202505090043).

### 5.1. Methods

To test the effectiveness of the system in intervening with office users’ behavior, this study compared changes in users’ sitting behaviors before and after wearing the experimental garment in a real-world environment. The effectiveness of the garment system was analyzed through objective data, and furthermore, user satisfaction was assessed through subjective ratings. The test selected eight sedentary office women aged 20–30 years with a height of 160 ± 5 cm and a weight of 48 ± 3 kg. Additionally, two experimental observers were selected to record the entire behavior of the users, specifically the time each sitting posture was maintained and the time points at which the sitting posture changed. The experiment was divided into two phases, each lasting 90 min. In phase 1, the user wore their own clothing without any feedback and performed the computerized task. At the end of phase 1, the user was required to get up and rest for 20 min, followed by phase 2, in which the user wore the experimental clothing and turned on the recognition and feedback system. At the end of the experiment, users were invited to fill out a subjective questionnaire regarding their satisfaction with the posture correction smart garment system. The questionnaire used a five-point Likert scale to measure satisfaction, where 1 stood for “very dissatisfied”, 2 for “not very satisfied”, 3 for “average”, 4 for “quite satisfied”, and 5 for “very satisfied.” The questionnaire collected users’ feedback in six areas: aesthetics, comfortableness, usability, responsiveness, intervention, and feedback.

### 5.2. Results

The results are divided into two parts: (1) objective data, which show and compare the number of times the user changed their sitting posture, verifying the effectiveness of the garment system’s intervention on sitting behavior, and (2) subjective rating data, which reflect the user’s overall feelings about the garment system.

**Figure 9:**
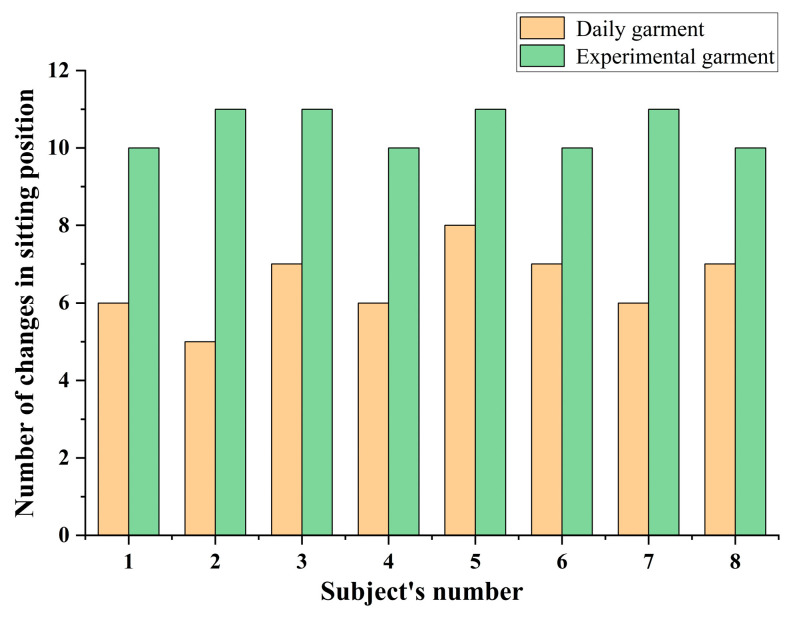
Comparison of the number of changes in users’ sitting postures in the before and after phases.

Firstly, the objective data were analyzed by recording the number of sitting posture changes during the two phases, where users wore daily clothing and experimental clothing. The results are shown in Figure 9. A paired-sample *t*-test was performed using the IBM SPSS Statistics 25 tool, and the results revealed that the number of sitting posture changes while wearing the daily clothing was significantly lower than the number of changes while wearing the experimental clothing (*t* = −8.79, *p* = 0.002 < 0.05), which indicates that the vibration feedback intervention while wearing the experimental clothing was effective in modifying the user’s sitting behavior and achieving dynamic sitting.

Secondly, the subjective scoring data were analyzed, and the results of the specific satisfaction questionnaire are shown in Table 4. The mean score of the questionnaire was 4.375, with a standard deviation of 0.49, indicating that users were very satisfied with the overall appearance of the garments. They also recognized the ease of use of the entire system and the way the feedback was provided. The lowest score was for the system’s intervention. Some users indicated that the vibration feedback was less noticeable when they were focused on their work, especially in the reclined position, because their backs were leaning against the backrest. This caused them to ignore the vibration reminder while concentrating on their tasks, which affected the intervention of the system. In the future, different vibration frequencies could be set for different sitting postures, particularly for leaning backward, to enhance the intensity and frequency of the vibration.

In summary, based on the analysis of both objective and subjective data, it can be concluded that the smart garment designed in this study effectively cultivates users’ behavioral awareness of the “dynamic sitting reminder”. The quantitative assessment of subjective data also demonstrates the practical value and application potential of the smart garment.

## 6. Discussion and Future Work

Overall, the sedentary office users who participated in the trial expressed a high level of satisfaction with the concept of the smart garment. They believe that the system’s posture reminder mode provides a better experience than existing smart posture products on the market. Li [21] designed the posture garment system to address the same problem as existing smart posture products, namely, that they generate vibration reminders once they detect poor sitting posture. Since it is impossible for people to maintain an upright sitting position all the time, these frequent vibration reminders often interrupt their thoughts during office hours, making them feel very disturbed. The system designed in this paper solves the above-mentioned pain points of sedentary office users through a reasonable reminder mode, and the system integrates the concepts of “dynamic sitting” and behavioral intervention into the design of the feedback mode, which is also a major innovation in the feedback mode settings of our system compared to other systems. Users also noted that the multi-sensory combination of tactile and visual feedback modes further enriched their experience. In work mode, the system can directly remind users through tactile vibration, while in non-work mode, users can also check their sitting posture and health data directly through the computer interface. This feedback mode is more intuitive and better suited to the user’s working conditions than the system developed by Ferdews Tlili [22], which uses phone vibration and screen notifications. In addition, Drashti Sikligar [19] and Yang [20] have designed clothing styles where the sensors are exposed on the outside, which greatly reduces users’ willingness to wear them. On the other hand, the smart garment style designed in this paper is simple and practical, making it very suitable for the current tastes of young people, and more users expressed their willingness to use the garment as daily wear for travel and office settings. The aesthetics and comfort were well received by the wearers, which enhanced their willingness to wear the garment.

However, our work also has several limitations. (1) Although textile sensors have been used in garments in order to achieve flexibility, the integration of other hardware is still insufficient and limited by the existing technical conditions, and the volume of electronic components, such as controllers, affects the fit and comfort of the garment. (2) The standardized haptic feedback mechanism fails to take individual differences into full consideration because different people actually have different perceptions of haptic vibration, and the fixed vibration frequency and time set by the system cannot meet the requirements of all people. (3) The data visualization function needs to be improved.

Future research should (1) adopt FPC flexible circuit board and inkjet printing wire technology to realize the integration of textiles so as to realize the flexibility of the garment; (2) further upgrade the function of the feedback mode, develop the haptic feedback system that can be adjusted individually, support the user to customize the vibration frequency and vibration time, and set different vibration modes; and (3) support the development of a mobile APP, providing real-time sitting posture monitoring, health data analysis, and other value-added functions to build a complete health management ecology. In addition, we should enhance the interactivity between the garment and the user, guiding them to get up and engage in physical activities, thereby truly achieving active health management.

## 7. Conclusions

In this paper, we propose a smart garment based on textile sensors that is capable of providing dynamic sitting posture reminders for sedentary office users, and we believe that this garment has important application value in sitting behavior intervention. Based on the analysis of the garment system requirements, we have proposed the entire design of the smart garment, including the electronic system and the design of the garment style structure, which effectively enhances both the comfort and aesthetics of the garment. In addition, the garment was worn to collect the user’s sitting posture data. Four algorithms were then used to compare the accuracy of sitting posture classification and recognition. The results demonstrated that the random forest algorithm had the best performance in terms of recognition accuracy. Importantly, the system is equipped with two feedback modes. In addition to the common visual feedback, which intuitively reflects the user’s sitting behavior and records sitting data, the system also features specially designed haptic vibration feedback based on the “Dynamic Sitting” concept for office scenarios. This mode reminds sedentary office users to change their sitting posture without disrupting their work. The results from the evaluation tests also confirmed the effectiveness of the garment system in intervening with the user’s behavior.

Future research will focus on enhancing the comfort of the garment and improving the functionality of the garment system. We hope that this system will be applicable not only to sedentary office workers but also to teenagers and students, helping every user to monitor and prevent spinal health issues, thereby reducing the health risks caused by sedentary activities in future society. We will continue to explore and optimize the design of the system, with the hope that it can be integrated with medical technology and extended for use in future wearable products for the spine or other bone health monitoring and rehabilitation.

## Figures and Tables

**Table 1:** Algorithms, parameters, and accuracy of the training models.

Number	Model	Parameters	Accuracy
1	K-Nearest Neighbor (KNN)	K = 1	94.32%
2	Support Vector Machine (SVM)	RBF, C = 10, gamma = 0.1	93.86%
3	Decision Tree	Max_depth = 10, Min_samples_leaf = 1, Min_samples_split = 2	81.27%
4	Random Forest	Max_depth = 20, Min_samples_leaf = 1, Min_samples_split = 2	95.62%

**Table 2:** Accuracy and standard deviation of ten-fold cross-validation for each model.

Number	Model	Mean Accuracy	Standard Deviation
1	K-Nearest Neighbor (KNN)	93.12%	0.82%
2	Support Vector Machine (SVM)	92.67%	1.43%
3	Decision Tree	78.36%	1.67%
4	Random Forest	94.93%	0.45%

**Table 3:** “Dynamic sitting” vibration pattern.

Type of Sitting Position	Posture Hazard Rating	Reminder Interval
Leg crossed	Serious	5 min
Sitting with one’s back bent, sitting forward, leaning backward, and other sitting postures	Medium	10 min
Sitting upright	Low	15 min

**Table 4:** The score of the Likert scale.

Evaluation Category	Question	Evaluation Projects	Average	Average Score of Questions	Standard Deviation
Aesthetics	Q1	Style design	5	4.77	0.00
	Q2	Color and fabric	4.54		0.50
Comfortableness	Q3	Electronic component arrangement and circuit design	4.31	4.35	0.46
	Q4	Overall feeling of wearing	4.38		0.49
Usability	Q5	The garment system is easy to operate and can be easily mastered by the user	4.6	4.6	0.74
Responsiveness	Q6	Overall responsiveness of the clothing system, able to respond quickly to user commands	4.2	4.2	0.46
Intervention	Q7	Garment systems can effectively intervene in users’ sitting behavior	3.8	3.8	0.53
Feedback	Q8	Feedback in a way that does not interfere with the user’s work	4.6	4.53	0.52
	Q9	Visual interface data are displayed correctly	4.2		0.76
	Q10	Visual interface design	4.8		0.52
All				4.375	0.49

## Data Availability

The data presented in this study are available upon request from the corresponding author due to privacy as this study is part of an ongoing graduate project.
